# The cGAS-STING pathway in traumatic brain injury: mechanisms of neuroinflammation and therapeutic strategies

**DOI:** 10.3389/fimmu.2026.1987328

**Published:** 2026-10-30

**Authors:** Bing Chen, Duochang Tu, Rong Zhang, Chuang Nie, Quanxing Shi, Cheng-teng Jiang, Feiyi Sun, Xiangrong Pu, Li Zhang, Qiang Zhou, Jian-wen Gu

**Affiliations:** 1 Department of Clinical Laboratory, The Second Hospital of Anhui Medical University, Anhui, China; 2 The Ninth Medical Center of Chinese PLA General Hospital, Beijing, China; 3 Air Force Special Medical Center, Beijing, China

**Keywords:** cGAS-STING, mitochondrial DNA, neuroinflammation, therapeutic strategies, traumatic brain injury

## Abstract

Traumatic brain injury (TBI) remains a leading cause of mortality and long-term disability worldwide, and no pharmacological therapy interrupts the deleterious secondary injury cascade. Damage-associated molecular patterns (DAMPs), particularly mislocalized nuclear and mitochondrial DNA, drive self-sustained neuroinflammation by activating the cytosolic DNA-sensing cGAS–STING pathway to trigger type-I interferon and NF-κB-dependent transcription. This review summarizes cGAS-STING molecular features, cell-specific activation, temporal kinetics and therapeutic strategies in TBI. Genetic studies from focal and diffuse injury models demonstrate that cGAS-STING ablation lessens lesion volume, mitigates neuroinflammation and preserves cognitive function, with microglia acting as the predominant cellular mediator. Clinical observations show that cell-free mitochondrial DNA, a major cGAS ligand, is markedly elevated in severe TBI patients’ cerebrospinal fluid and serum, correlating with injury severity and prognosis, while aging amplifies pathway activation. Positioned at the intersection of DAMP sensing, glial reactivity, cell-death signaling and chronic neurodegeneration, cGAS-STING represents a highly promising therapeutic target for post-traumatic neuroinflammation.

## 1 Introduction

### 1.1 The critical role of neuroinflammation in the pathogenesis of traumatic brain injury

Traumatic brain injury (TBI) is a leading cause of mortality and long-term disability across all age groups, imposing a substantial disease burden worldwide. Epidemiological data indicate that TBI results in approximately 2.9 million hospitalizations and 55 000 deaths annually, with the most frequent causes such as road traffic collisions, falls, sports injuries, blast exposure, and violent or abusive trauma (1, 2). Clinically, TBI is defined as brain dysfunction or structural damage induced by external mechanical forces, manifesting as neurological deficits including altered consciousness, memory impairment, cognitive slowing, and disorientation (3–5). Pathologically, TBI injury progression is categorized into two stages: primary and secondary injury. Primary injury refers to immediate mechanical damage occurring at the moment of trauma, including axonal shearing, cerebral contusion, blood–brain barrier (BBB) disruption, cerebral edema and hemorrhage, which further triggers a sustained, non-mechanical and multifactorial secondary injury cascade (6). This protracted secondary pathological process lasts from hours to even years, involving excitotoxicity, mitochondrial dysfunction, oxidative stress, neuroinflammation, endoplasmic reticulum stress and neuronal apoptosis, and ultimately leads to intractable secondary complications such as elevated intracranial pressure, increased seizure risk and chronic neuroinflammation. These sequelae are closely associated with poor neurological prognosis and long-term cognitive and affective dysfunction in survivors (7, 8). As a key delayed and progressive pathological consequence of primary TBI injury, neuroinflammation is a major driver of post-traumatic neurodegeneration and white matter degeneration (9, 10). It arises from the excessive activation of the central microglial innate immune system, combined with the activation of reactive astrocytes and infiltration of peripheral immune cells, triggering the massive release of proinflammatory cytokines and chemokines (11, 12). Notably, post-traumatic neuroinflammation exhibits dual regulatory effects of repair and destruction; however, excessive and persistent inflammatory responses will drive progressive brain tissue loss and continuous functional decline (13).

### 1.2 Innate DNA sensing: a paradigm shift in TBI neuroinflammation

A conceptual breakthrough in innate immunity over the past fifteen years has reshaped our understanding of post-traumatic neuroinflammation. The innate immune system detects cellular damage through pattern recognition receptors (PRRs) that sense damage-associated molecular patterns (DAMPs)—endogenous molecules such as nuclear and mitochondrial DNA released from injured cells (14, 15). Among these sensing systems, the cytosolic DNA-sensing cyclic GMP-AMP synthase (cGAS)–stimulator of interferon genes (STING) pathway has emerged as a central signaling hub. STING was first identified as an endoplasmic reticulum adaptor of innate immune signaling in 2008 (16, 17), and its upstream sensor cGAS, together with the second messenger cyclic GMP-AMP (cGAMP), was defined in 2013 as the core machinery that transduces cytosolic double-stranded DNA into type I interferon and nuclear factor-κB (NF-κB) transcriptional programs (15, 18, 19).

Since mitochondrial stress, nuclear membrane rupture and cell death represent ubiquitous pathological characteristics of traumatized brain tissue, the cGAS–STING pathway serves as a convergence node for diverse DAMPs released following TBI (20, 21). The involvement of cGAS–STING in neuroinflammation and neurodegeneration has been well documented in aging and multiple disease models (22–25). Importantly, direct evidence linking this pathway to TBI has emerged only in recent years. In 2018, STING-dependent type I interferon signaling was reported to mediate harmful neuroinflammation after experimental traumatic brain injury (26, 27). Subsequent studies further identified that the NLRX1–cGAS–STING signaling axis coordinates type I interferon responses upon brain trauma, alongside observations confirming the translocation of mitochondrial DNA into the cytosol of injured cortical cells (28, 29). A rapidly expanding series of genetic and pharmacological studies through 2024–2025 now places this pathway at the core of the post-traumatic inflammatory cascade (23, 30–34).

Several recent reviews have surveyed cGAS–STING signaling in neurodegeneration and brain aging broadly (22–25), and others have highlighted mitochondrial DNA as a general driver of sterile neuroinflammation (35). The present review differs from these articles in scope: it focuses specifically and comprehensively on TBI, integrating the pathway’s molecular architecture, cell-type-resolved and temporally resolved activation, crosstalk with regulated cell-death programs, and the full spectrum of genetic and pharmacological evidence in both focal and diffuse injury paradigms, together with the emerging human biomarker data that connect the pathway to injury severity and prognosis in patients.

### 1.3 Rationale for targeting cGAS–STING in TBI

Within the multi-receptor landscape of post-traumatic neuroinflammation, the cGAS–STING pathway occupies a distinctive position: whereas Toll-like receptors (TLRs) and nucleotide-binding oligomerization domain (NOD)-like receptors (NLRs) survey extracellular and vacuolar compartments, cGAS directly monitors the cytosol, the compartment into which trauma channels mitochondrial DNA (mtDNA), chromatin fragments and neutrophil extracellular traps (NET)-derived DNA (36, 37). Cytosolic DNA sensing therefore functions as a convergence point that integrates the diverse DAMP species generated by mechanical injury into unified type I interferon and NF-κB outputs, providing a mechanistic rationale for the pathway’s prominence in post-traumatic neuroinflammation and for its candidacy as a therapeutic target (38, 39). This review critically synthesizes the molecular architecture, cell-type-resolved deployment, temporal dynamics, crosstalk with cell-death programs, and therapeutic tractability of cGAS–STING signaling in TBI, with the goal of defining a roadmap for translational development.

## 2 The cGAS–STING signaling pathway

### 2.1 Molecular mechanism of cGAS activation

The cGAS-STING pathway constitutes the principal cytosolic route by which eukaryotic cells detect mislocalized double-stranded DNA (dsDNA) and convert that detection into an innate immune transcriptional program (15) (see **Figure 1**). cGAS (MB21D1) was purified from cytosolic extracts and shown to undergo a conformational switch upon binding dsDNA that enables its catalytic pocket to convert adenosine triphosphate (ATP) and guanosine triphosphate (GTP) into cGAMP, a second messenger that is both necessary and sufficient to engage STING (15). Structural and biochemical characterization revealed the physiological product to be 2’3’-cGAMP, a cyclic dinucleotide distinguished from bacterial cyclic di-GMP and cyclic di-AMP by a noncanonical 2’-5’ phosphodiester linkage (G2’-5’pA3’-5’-p); this isomer binds the ligand-binding domain of the STING dimer with high affinity and can additionally be transmitted to bystander cells through gap junctions (18, 19). Because cGAS is activated by dsDNA irrespective of sequence, the identity of the ligand-bearing compartment—rather than the sensor itself—determines when and where the pathway is activated (40).

**Figure 1:**
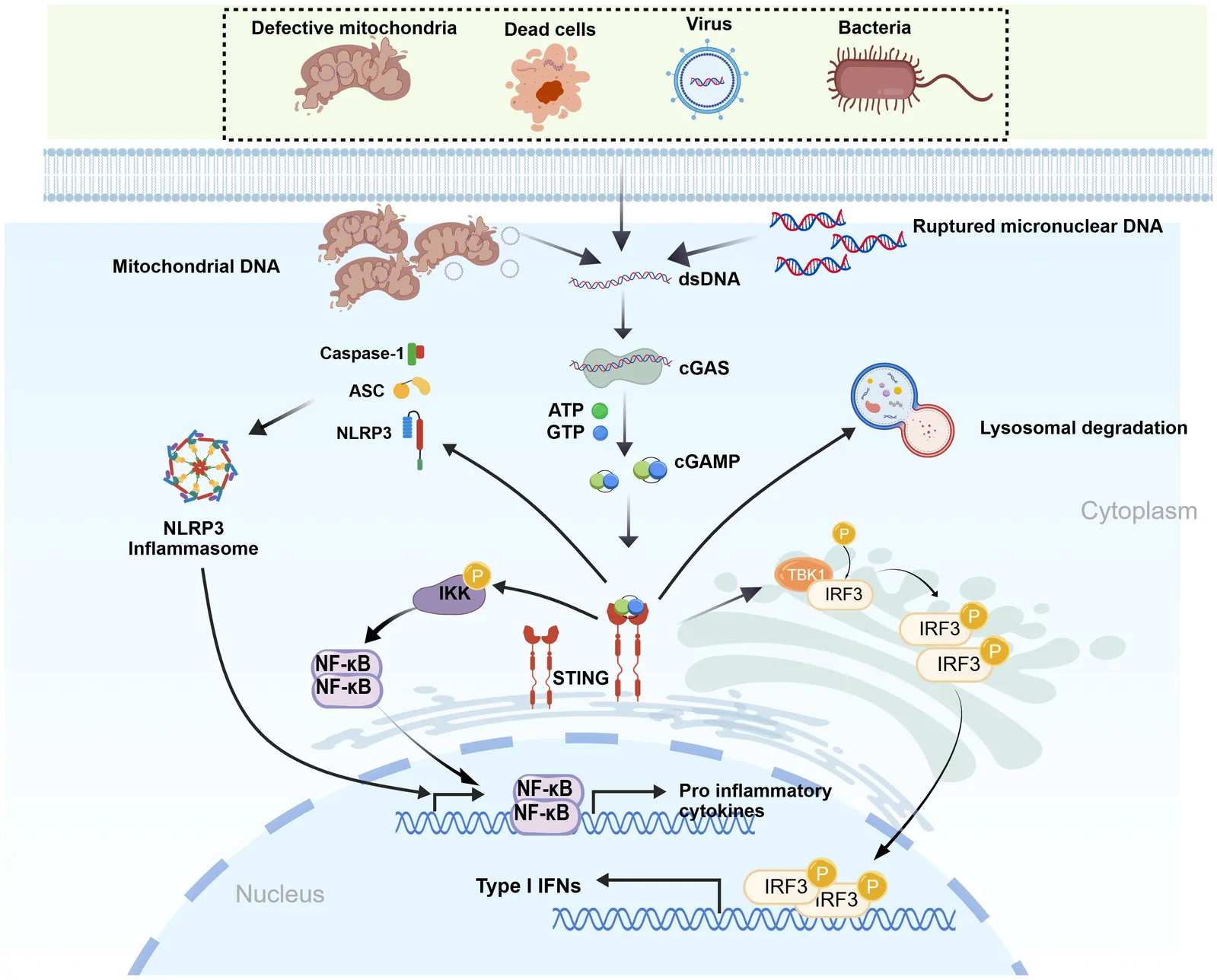
The canonical cGAS–STING signaling axis and its cellular outputs. Cytosolic double-stranded DNA derived from mitochondrial DNA, ruptured micronuclei, cytoplasmic chromatin fragments and foreign pathogenic microorganism bind and activates cyclic GMP–AMP synthase (cGAS), which synthesizes the second messenger 2’3’-cGAMP. cGAMP binding drives endoplasmic reticulum (ER)-resident STING to translocate via the ER–Golgi intermediate compartment to the Golgi, where palmitoylation at Cys88/91 licenses its clustering and signaling. At this platform, TBK1 phosphorylates STING at Ser366 and recruits IRF3 to induce type I interferon, while a parallel IKK–NF-κB arm induces TNF-α, IL-6 and IL-1β. Beyond transcriptional outputs, STING trafficking initiates autophagy—the pathway’s primordial function and termination mechanism—and a lysosomal cell death program that acts upstream of NLRP3 inflammasome activation and pyroptosis, integrating the axis with broader cell death and neuroinflammatory networks (64).

In humans, cGAS belongs to the nucleotidyl-transferase (NT) family and is encoded by the MB21D1 gene. Human cGAS consists of a single NT domain and two primary DNA-binding regions: a flexible N-terminal domain and a structured C-terminal region that includes both NT and Mab-21 domains (41, 42). The catalytic C-terminal domain contains a zinc-binding motif essential for recognizing cytoplasmic DNA; disruption of this region prevents cGAS from detecting DNA (41). In the absence of dsDNA, cGAS exists as a catalytically inactive monomer. Upon binding to dsDNA, conformational changes occur in the catalytic center, encouraging dimerization of cGAS molecules and leading to activation (43).

The subcellular localization of cGAS remains a subject of ongoing investigation, with evidence supporting its presence in the nucleus, cytosol, and plasma membrane. Recent studies report that the majority of cGAS resides in the nucleus prior to activation, regardless of the cell’s proliferative status, and secure nuclear tethering through its NTase domain is essential for preventing autoreactivity (44). During the G2/M phase, cGAS assumes a unique state characterized by eight residues within its disordered N terminus, which helps inhibit erroneous activation (45).

### 2.2 STING activation and downstream signaling: TBK1–IRF3 and NF-κB axes

STING (also designated TMEM173, MITA, MPYS, or ERIS) is an ER-resident transmembrane protein of 379 amino acids. It possesses an N-terminal four-helical transmembrane domain that assembles into a domain-swapped homodimer, and a cytosolic region comprising a globular ligand-binding domain (LBD) plus a flexible, intrinsically disordered C-terminal tail (CTT) (16, 46). The N-terminal domain, positioned across the ER membrane, consists of four transmembrane domains that anchor STING to the ER membrane. The C-terminal domain includes a globular CTT and a ligand-binding domain (LBD), both essential for STING’s activation and function (46).

Conformational switch and higher-order oligomerization of the ER-resident STING dimer, exits the ER, and assembles a signaling platform that couples TANK-binding kinase 1 (TBK1) recruitment to interferon regulatory factor 3 (IRF3) phosphorylation through defined linear motifs. A conserved C-terminal PLPLRT/SD motif of STING directly recruits and activates TBK1 (47, 48); TBK1 in turn phosphorylates STING at Ser366 within a pLxIS motif, creating a phospho-docking site for IRF3, which is then phosphorylated, dimerizes, and translocates to the nucleus to drive IFN-β and interferon-stimulated gene (ISG) expression (49, 50). In parallel, STING engages the inhibitor of κB kinase (IKK)-NF-κB module; in myeloid cells this arm depends on functional redundancy between TBK1 and IKKϵ, and it drives transcription of the pro-inflammatory cytokines tumor necrosis factor-α (TNF-α), interleukin-6 (IL-6), and interleukin-1β (IL-1β), together with chemokines such as C-X-C motif chemokine ligand 10 (CXCL10) and CCL5 (32, 51, 52). The dual IFN-I/NF-κB output of this cascade—precisely the cytokine axis (TNF-α, IL-6, IL-1β, IFN-β) that is amplified in the injured brain—positions cGAS-STING as a central node in post-traumatic neuroinflammation (32, 51).

STING activation is inseparable from membrane trafficking. cGAMP binding obliges STING to translocate from the ER through the ER-Golgi intermediate compartment (ERGIC) to the Golgi; blocking ER-to-Golgi traffic with brefeldin A or by disrupting COP-II/Sar1 machinery abolishes signaling, and ER-retained STING is inactive (53, 54). The physiological importance of this itinerary is underscored by gain-of-function mutations causing STING-associated vasculopathy with onset in infancy (SAVI), which constitutively exit the ER in the absence of ligand (54, 55). Upon arrival at the Golgi, STING undergoes palmitoylation at the transmembrane-proximal cysteines Cys88 and Cys91, a post-translational modification required for its clustering into puncta and for productive TBK1 recruitment; palmitoylation inhibitors such as 2-bromopalmitate block STING signaling (56–58). This cysteine dependence provided the mechanistic foundation for covalent STING antagonists—notably H-151 and C-178—that alkylate Cys91, lock STING in an inactive state, and are now being explored in neuroinflammatory settings (33, 59, 60).

### 2.3 Negative regulation and signal termination mechanisms

Signal termination is equally choreographed and occurs through at least two complementary mechanisms. First, cyclic dinucleotide stimulation activates UNC-51-like kinase 1 (ULK1/ATG1), which phosphorylates STING at Ser366 and thereby suppresses IRF3 activity, imposing a negative-feedback brake that prevents sustained innate signaling (61). Second, the same trafficking route that activates STING also delivers it for degradation: ERGIC-derived membranes carrying activated STING serve as platforms for LC3 lipidation, and the resulting autophagosomes ferry STING—together with engulfed DNA or virions—to lysosomes for destruction (62). Autophagic clearance thus simultaneously disposes of the ligand, the adaptor, and the signaling complex, coupling the initiation of STING signaling to its own extinction (61, 62). These termination mechanisms are of direct relevance to TBI, where excessive STING trafficking after trauma may overload the lysosomal system, converting a homeostatic clearance route into membrane permeabilization that feeds inflammasome activation (63).

## 3 The cGAS–STING pathway in TBI: activation and downstream neuroinflammatory cascades

### 3.1 Cytosolic DNA as DAMPs: sources of endogenous ligands

#### 3.1.1 Mitochondrial DNA release and oxidative stress

Mitochondrial DNA is a dominant endogenous agonist in injured tissue (see **Figure 2**). During apoptosis, oligomerized BAK and BAX form macropores in the mitochondrial outer membrane; live-cell lattice light-sheet microscopy demonstrated that the inner membrane herniates through these widening pores, releasing matrix contents including mtDNA into the cytosol (65). This process, termed mitochondrial inner membrane permeabilization (MIMP), is a mechanistically distinct step from mitochondrial outer membrane permeabilization (MOMP) and constitutes the physical route by which mtDNA gains access to cytosolic cGAS (65, 66). Critically, apoptotic caspases normally silence this mtDNA-driven inflammation: BAX/BAK-mediated MOMP releases mtDNA that activates cGAS-STING-IFN signaling, but effector caspases-3 and -7 cleave cGAS and IRF3 and thereby suppress the response; when caspases are inhibited or deleted, apoptotic cells become strongly immunostimulatory and drive lethal type I IFN-dependent inflammation *in vivo (*20, 21, 67, 68). This finding is directly relevant to TBI, where the balance between caspase activation and caspase-deficient death modalities (necroptosis, pyroptosis) will determine whether dying neural cells amplify or dampen cGAS-STING output (69, 70).

**Figure 2:**
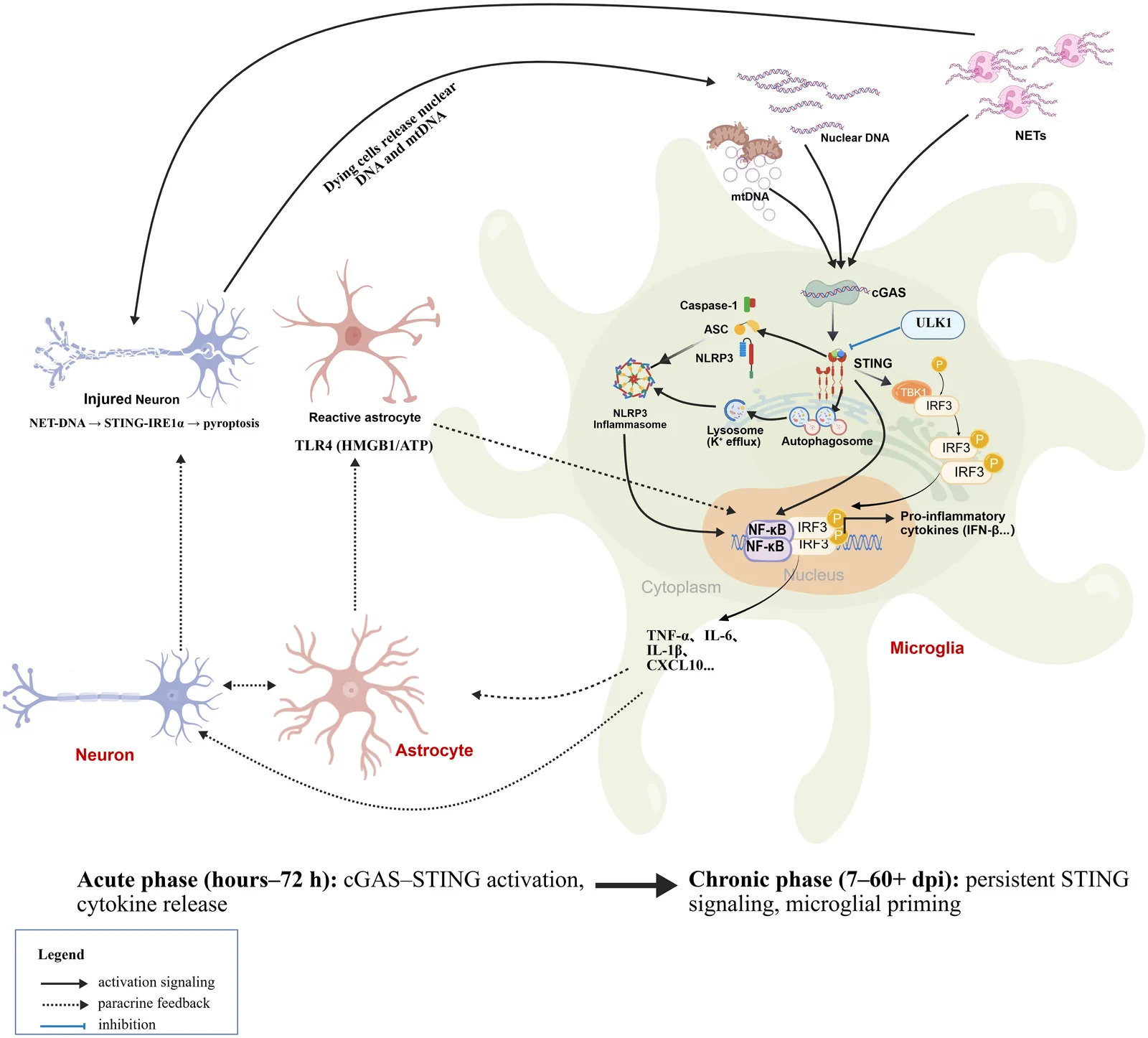
The cGAS–STING mechanisms after traumatic brain injury. Schematic diagram illustrating the canonical cGAS–STING signaling cascade in microglia following traumatic brain injury. After TBI, damaged neurons release mitochondrial DNA (mtDNA), and neutrophil extracellular traps (NETs) provide extracellular DNA fragments. These cytosolic double-stranded DNAs are sensed by cGAS in microglia, which catalyzes the production of the second messenger cGAMP. cGAMP binds and activates STING, which subsequently recruits TBK1 to phosphorylate IRF3. Phosphorylated IRF3 translocates into the nucleus to drive the transcription of pro-inflammatory cytokines. Meanwhile, activated STING initiates the IKK–NF-κB signaling axis to further promote inflammatory gene expression. In addition, STING signaling facilitates NLRP3 inflammasome assembly, which activates caspase-1 and amplifies inflammatory responses in microglia.

Fritsch et al. provided the first direct ligand evidence in TBI, detecting mtDNA in the cytosol of injured cortical cells acutely after controlled cortical impact (CCI)—a canonical trigger for cGAS (28, 34). Two human biomarker studies establish clinical relevance: in pediatric severe TBI, CSF mtDNA was elevated nearly 70-fold over controls (1.10×10^5^ ± 2.07×10^5^ vs 1.63×10³ ± 1.80×10³ copies/μL), tracked with outcome (1.63×10^5^ copies/μL in non-survivors or severe disability vs 5.05×10^4^ in survivors; p < 0.0001), correlated with HMGB1 (ρ = 0.574), and yielded an ROC AUC of 0.77 (95% CI 0.51-0.90) for 6-month outcome (71, 72). In adults sampled within 48 h, cell-free mtDNA was elevated in both serum (p < 0.0001) and CSF (p = 0.0007) of severe TBI patients, and across the combined TBI/SAH cohort, cell-free mtDNA correlated inversely with admission Glasgow Coma Scale score (Spearman ρ = -0.74, p = 0.0025) and positively with CSF IL-6 (ρ = 0.66, p = 0.009), linking the DAMP to injury severity and downstream inflammation (73). These biomarker findings should nevertheless be interpreted cautiously: both studies were small, single-center and exploratory, with heterogeneous patient selection and assay methodologies, and the moderate area under the receiver-operating curve indicates limited standalone diagnostic performance. Large, multicenter prospective studies with standardized quantification of cell-free mtDNA will be required before this DAMP can be used clinically as a prognostic or patient-stratification biomarker.

#### 3.1.2 Nuclear DNA leakage from injured neurons

Nuclear-derived DNA species provide a second ligand pool. Micronuclei arising from chromosome missegregation or unrepaired DNA damage undergo irreversible nuclear envelope rupture during interphase, and cGAS accumulates within ruptured micronuclei as cells progress through mitosis, linking chromosomal instability to innate immune activation (74). In senescent cells, cytoplasmic chromatin fragments (CCFs)—extranuclear chromatin marked by γH2AX and H3K27me3—bud from the nucleus and activate cGAS-STING to sustain the senescence-associated secretory phenotype (SASP) and paracrine senescence; genetic ablation of cGAS or STING blunts SASP both *in vitro* and *in vivo* (75, 76). A related mechanism operates in senescence through “minority MOMP” (miMOMP), in which sublethal apoptotic stress permeabilizes only a small subset of mitochondria; mtDNA efflux through BAX/BAK macropores under these conditions activates cGAS-STING and drives the SASP without killing the cell, and pharmacological blockade of MOMP in aged mice reduces sterile inflammation and improves healthspan (77, 78). These senescence- and aging-associated outputs are of particular consequence for TBI, in which injury-induced cellular senescence and an aged immune milieu amplify and prolong cGAS-STING-driven inflammation well beyond the acute phase (79).

#### 3.1.3 Neutrophil extracellular traps

Extracellular self-DNA can feed the pathway: the DNA scaffold of neutrophil extracellular traps is recognized by cGAS in phagocytes, triggering cGAMP production, STING activation, and IFN-I release (80, 81). NETs accumulate in the brains of TBI patients and correlate with intracranial pressure and neurological deterioration; in mice, NET-derived DNA activates neuronal STING, which engages the ER-stress sensor IRE1α to drive inflammasome-dependent pyroptosis (82–84). DNase-1 digestion of NET DNA or PAD4 inhibition blunted this cascade, and protection was reversed by exogenous 2’3’-cGAMP, establishing STING as the relay between extracellular self-DNA and inflammasome execution (84, 85).

### 3.2 Cell-type-specific activation

#### 3.2.1 Microglia: the principal IFN-I producers

Bone-marrow chimera experiments refined the cellular source of protection after CCI. Reciprocal wild-type/STING^−/−^ chimeras showed that loss of STING in either microglia or peripheral hematopoietic cells alone conferred intermediate protection, whereas combined deficiency reduced 24-h lesion volume by approximately 50% (≈2.3 vs ≈4.8 mm³; F(3,25)=12.12, p=4.3×10^−5^), together with markedly reduced neutrophil infiltration and IgG deposition (32). Transcriptomics at 2 h showed that microglia drive an early STING-dependent pro-inflammatory program, including IL-1β, that shapes later leukocyte recruitment (34). After diffuse midline fluid percussion injury (mFPI), inducible microglia-specific STING ablation attenuated 85% of the 237 cortical differentially expressed genes induced by mFPI, prevented approximately half of the TBI-induced neuronal transcriptomic changes (including suppression of CREB and synaptogenesis programs), reduced plasma neurofilament light chain, and rescued cognition (34, 86). Critically, global interferon-α/β receptor 1 (IFNAR1) knockout did not protect cognition or cortical inflammation at 7 dpi in this diffuse paradigm (34), in contrast to the protection conferred by hematopoietic IFNAR1 ablation after CCI (87)—indicating that microglial STING acts partly through IFNAR1-independent effectors in diffuse injury.

#### 3.2.2 Astrocytes: amplifiers of inflammatory signals

STING protein co-localized prominently with GFAP^+^ astrocytes and neurons in the acute phase after CCI, implicating astroglial signaling (32). Global STING knockout attenuated astrocyte reactivity acutely (32), although cortical astrogliosis after mFPI is independent of microglial STING (30), suggesting model-dependent contributions.

#### 3.2.3 Infiltrating peripheral immune cells and neuronal intrinsic responses

Peripheral hematopoietic STING signaling is essential for neutrophil and monocyte infiltration (32). Neutrophils, through NETs formation, provide exogenous DNA that activates neuronal STING (26, 32). Neurons themselves engage the pathway through multiple routes: injured neurons release mtDNA into the cytosol, engaging canonical cGAS-STING-TBK1-IRF3 signaling, while ER stress activates a noncanonical, cGAS-independent PERK-STING route that drives neuronal IFN-β output (88). Neurons additionally receive NET-derived DNA, which triggers STING-IRE1α-dependent pyroptosis (26, 83).

Beyond the CNS-resident and infiltrating compartments described above, recent work indicates that cGAS–STING signaling in peripheral immune cells and CNS microglia act cooperatively to shape injury outcome. Using reciprocal bone-marrow chimeras and conditional knockouts, Fritsch et al. showed that loss of STING restricted to either microglia or peripheral hematopoietic cells conferred only intermediate protection after controlled cortical impact, whereas combined deficiency was required for maximal benefit, underscoring coordinated signaling between brain-resident and peripheral immune compartments. Whether gut–brain immune crosstalk—for example, trauma-associated dysbiosis or microbial DNA sensed in the enteric immune system—further contributes to the systemic and cerebral inflammatory trajectory after TBI remains an important open question and a promising direction for future investigation (38, 89).

### 3.3 Temporal dynamics: from acute to chronic phases

The temporal profile of pathway activation is biphasic and, at least for STING, remarkably persistent. STING and ISG expression rise within hours of CCI, wane, and re-emerge in a second wave (34), with robust protein-level induction of cGAS, STING, IFN-β, and STAT1 at 72 h in cortex and hippocampus (26, 90). Strikingly, at 60 dpi cortical STING (p = 0.0006), STAT1, IRF1, IRF7, CXCL10, ISG15, and IFI204 remained elevated while cGAS had normalized—implying a chronic phase of STING-dependent signaling that outlasts activation of the initiating sensor (26). Diffuse TBI shows an analogous trajectory: cortical STING protein and interferon-responsive microglia peak at 7 dpi, followed at 30 dpi by a primed microglial phenotype (Clec7a, Itgax, H2-eb1) that is STING-dependent and accompanied by persistent cognitive deficits (30). Functional data mirror this chronicity: IFN-β^−/−^ mice show improved motor function and recognition memory through 28 dpi and reduced lesion volume at that late time point (90). Together these studies suggest two therapeutic windows—acute blockade to limit secondary injury and sustained or delayed blockade to reverse microglial priming—and identify STING itself, rather than upstream cGAS, as the more durable target (30, 90).

Aging as an amplifier. Aging, the strongest biological modifier of TBI outcome, amplifies pathway activation. In aged (22-month) mice, mild CCI induced significantly greater cortical cGAS protein accumulation (young-TBI vs aged-TBI, p=0.0007), STAT1 phosphorylation (p=0.0035), and Ifnb1, Irf7, Ifi204, and Isg15 transcription than in young adults, with significant age×injury interactions (79, 91). After diffuse mFPI, aging similarly amplified gliosis and interferon-associated inflammation, and in young adult mice, pretreatment with the STING agonist DMXAA further augmented the injury-induced STING/IFN transcriptional signature—consistent with heightened STING activation in young adults being sufficient to reproduce key features of the aged inflammatory phenotype (79). These findings align with evidence that cGAS-STING drives age-related neuroinflammation even in the absence of trauma (92) and are consistent with age-dependent mitochondrial fragility lowering the threshold for ligand release after injury. Sex as a biological variable. Sex differences in TBI epidemiology, pathophysiology and outcome are well documented (93), yet the cGAS–STING studies reviewed here were performed almost exclusively in male rodents—for example, the aging study of Barrett et al. (91)and the chimera study of Fritsch et al. (34) used male mice only. Sex-dependent differences in mitochondrial quality control, baseline interferon tone and estrogen-mediated immunomodulation may render the female response to TBI distinct, and future studies should include both sexes with sex-stratified analyses to determine whether the therapeutic window and dosing of cGAS–STING inhibition differ between males and females.

### 3.4 Neuroinflammatory effectors: type I interferon responses, cytokine storms, and blood-brain barrier disruption

The transcriptional outputs of cGAS-STING translate into distinct neuroinflammatory phenotypes after TBI. The type I interferon response is a consistent feature of the post-traumatic neuroimmune response—interferon-β released by stressed neurons amplifies microglial activation and T-cell recruitment and promotes white-matter injury (70), and sustained interferon signaling enhances neuroinflammation that drives chronic neurodegeneration (26, 90). In parallel, the NF-κB arm drives TNF-α, IL-6, IL-1β, CXCL10, and CCL5 production (32, 51), constituting a cytokine storm that amplifies glial reactivity and recruits peripheral leukocytes. Notably, Evans Blue extravasation at 24 h did not differ between genotypes in one CCI paradigm, indicating that acute blood-brain barrier (BBB) leakage is not cGAS/STING-dependent in all models; however, subacute BBB disruption emerges through NET-driven STING-IRE1α/ASK1/JNK signaling, which disrupts endothelial tight junctions and cerebral perfusion (83).

### 3.5 cGAS–STING and regulated cell death: inflammasome-driven pyroptosis and crosstalk with apoptosis, necroptosis and ferroptosis

The mechanistic basis for STING-inflammasome coupling was defined in human myeloid cells, where cytosolic DNA-activated STING traffics to lysosomes, permeabilizes their membranes, and releases cathepsins; the resulting K^+^ efflux activates the NLRP3 inflammasome, caspase-1, and pyroptotic IL-1β release (63, 64). In parallel, STING-driven NF-κB—mediated redundantly by TBK1 and IKKϵ—transcriptionally primes NLRP3, caspase-1, and pro-IL-1β, so cGAS-STING supplies both the priming and the activation step of inflammasome assembly (94–96). In a rat model of severe TBI, the STING inhibitor C-176 attenuated NLRP3, cleaved caspase-1, and gasdermin-D (GSDMD) expression, reduced NLRP3-dependent pyroptosis and neuronal loss, and improved cognitive and affective outcomes; the protection was partially reversed by the NLRP3 activator nigericin, while the STING agonist ADU-S100 exacerbated pyroptosis and neurological deficits—placing NLRP3 downstream of STING in the injured brain (97). NET-derived DNA activates neuronal STING-IRE1α to drive NLRP1/caspase-1/GSDMD-dependent neuronal pyroptosis, reversible by DNase-1 or PAD4 inhibition (26, 85).

Apoptosis engages the pathway in a bidirectional manner. On one hand, BAX/BAK-mediated MOMP and MIMP release mtDNA that potently activates cGAS–STING; on the other hand, effector caspases-3 and -7 cleave and inactivate cGAS, MAVS and IRF3, so that apoptosis ordinarily proceeds without inflammatory output, whereas caspase-deficient death becomes strongly immunostimulatory (20, 21, 67, 68). Which apoptotic death is caspase-competent and which is not after TBI will therefore determine whether dying neural cells amplify or silence cGAS–STING output, a balance that remains to be mapped experimentally in the injured brain.

Necroptosis and ferroptosis also intersect with cytosolic DNA sensing. RIPK1/RIPK3/MLKL-mediated necroptotic membrane rupture releases DAMPs—including genomic and mitochondrial DNA—that can feed cGAS–STING activation, establishing a feed-forward loop between regulated necrosis and interferon-driven inflammation. In TBI specifically, NET-derived DNA aggravates blood–brain barrier disruption through ZBP1/FSP1-mediated ferroptosis (83) and iron-dependent lipid peroxidation with mitochondrial damage supplies an abundant pool of oxidized mtDNA ligands. More broadly, cGAS–STING has been linked to ferroptotic and necroptotic death programs in non-CNS models (64, 94); however, direct evidence in TBI remains scarce, and the relative contribution and temporal succession of each death program after injury warrant systematic investigation (98).

### 3.6 Autophagy homeostasis imbalance

STING’s relationship with autophagy is evolutionarily older than its relationship with interferon. Upon cGAMP binding, STING-containing ER-Golgi intermediate compartment membranes serve directly as platforms for LC3 lipidation, generating autophagosomes that deliver STING—and engulfed cytosolic DNA—to the lysosome (62). This induction is TBK1/IRF3-independent and is preserved in metazoans lacking the interferon system, identifying autophagy as the primordial output of the cGAS pathway (62). A second brake acts through the autophagy kinase ULK1, which phosphorylates activated STING (Ser366) to extinguish sustained IRF3 activity (61). After controlled cortical impact, wild-type mice accumulate LC3-II, p62, and LAMP2 in injured cortex—a signature of stalled autophagic flux with lysosomal stress—whereas STING-deficient animals show attenuated LAMP2 induction, consistent with restored flux (32). Excessive STING trafficking after trauma may thus overload the lysosomal system it engages, converting a homeostatic clearance route into the membrane permeabilization that feeds NLRP3 (32, 63). Enhancing autophagic quality control to starve cGAS of substrate offers an upstream strategy: the hormone FGF21 promoted AMPK-dependent mitophagy, prevented cytosolic mtDNA release, and thereby inhibited cGAS-STING in subarachnoid hemorrhage—a template directly relevant to TBI, where mitochondrial damage and mtDNA efflux are abundant (99).

### 3.7 Crosstalk with other PRRs and noncanonical activation

#### 3.7.1 TLRs/NLRs synergistically amplify inflammation

The secondary cascade is initiated and sustained by DAMPs that engage multiple PRRs simultaneously. High-mobility group box 1 (HMGB1) and extracellular ATP activate TLRs and purinergic/inflammasome pathways, while cytosolic DNA activates cGAS-STING (100–103). Within this multi-receptor landscape, cGAS-STING occupies a distinctive position as a cytosolic convergence point that integrates diverse DAMP species into unified type I interferon and NF-κB outputs (32, 38, 89).

#### 3.7.2 The PERK-STING axis: ER stress-mediated cGAS-independent activation

Neuronal ER-stress/PERK signaling activates STING-IRF3-IFN-β independently of cGAS after CCI and drives white-matter injury (104, 105). The PERK inhibitor GSK2656157 abrogated this noncanonical route, switching microglia toward an anti-inflammatory phenotype, reducing Th1-cell infiltration, preserving white-matter integrity on diffusion tensor imaging, and improving affective outcomes (105, 106). This cGAS-independent activation mechanism implies that post-traumatic STING signaling is not exclusively ligand-driven, and that combined blockade at multiple nodes may be required for maximal benefit.

## 4 Therapeutic strategies and translational prospects

### 4.1 Genetic and pharmacological evidence

#### 4.1.1 Protective effects of cGAS^−/−^ and STING^−/−^ models

Convergent genetic evidence across focal and diffuse rodent models demonstrates that pathway disruption is protective. Global STING^−/−^ mice developed significantly smaller lesions at 24 h after CCI, with reduced TNF-α, IL-1β, IFN-α/β, and IRF3 transcripts, attenuated astrocyte and microglial reactivity, and normalization of autophagy markers (36). Both cGAS^−/−^ and STING^−/−^ mice showed reduced lesion volumes and fewer TUNEL^+^ cells at 24 h, with protection persisting to 14 dpi, reduced CCL2 and CXCL10, and improved motor performance (34). Bone-marrow chimera experiments showed that combined microglial and hematopoietic STING deficiency reduced 24-h lesion volume by approximately 50% (34). After diffuse mFPI, global STING knockout abolished cognitive deficits at 7 and 30 dpi (107), and microglia-specific STING ablation rescued neuronal dysfunction (107).

#### 4.1.2 Small-molecule inhibitors: cGAS inhibitors, STING antagonists, and PERK inhibitors

The covalent STING antagonist C-176 provides the most direct TBI evidence (see **Figure 3**). A single intravenous dose (750 nmol) administered 30 min after CCI reduced cortical lesion area at 24 h, attenuated striatal Tnf-α, Il-1β, and Cxcl10 expression, and improved DigiGait parameters (33). In rat severe TBI, C-176 alleviated cognitive and emotional deficits, neuronal loss, glial activation, and NLRP3/caspase-1/GSDMD-mediated pyroptosis; protection was partially reversed by nigericin, and the STING agonist ADU-S100 worsened outcomes (97). C-176 also abolished NET/PAD4-driven neuronal pyroptosis and apoptosis (108, 109). However, C-176 is mouse-selective, limiting clinical development value (59).

**Figure 3:**
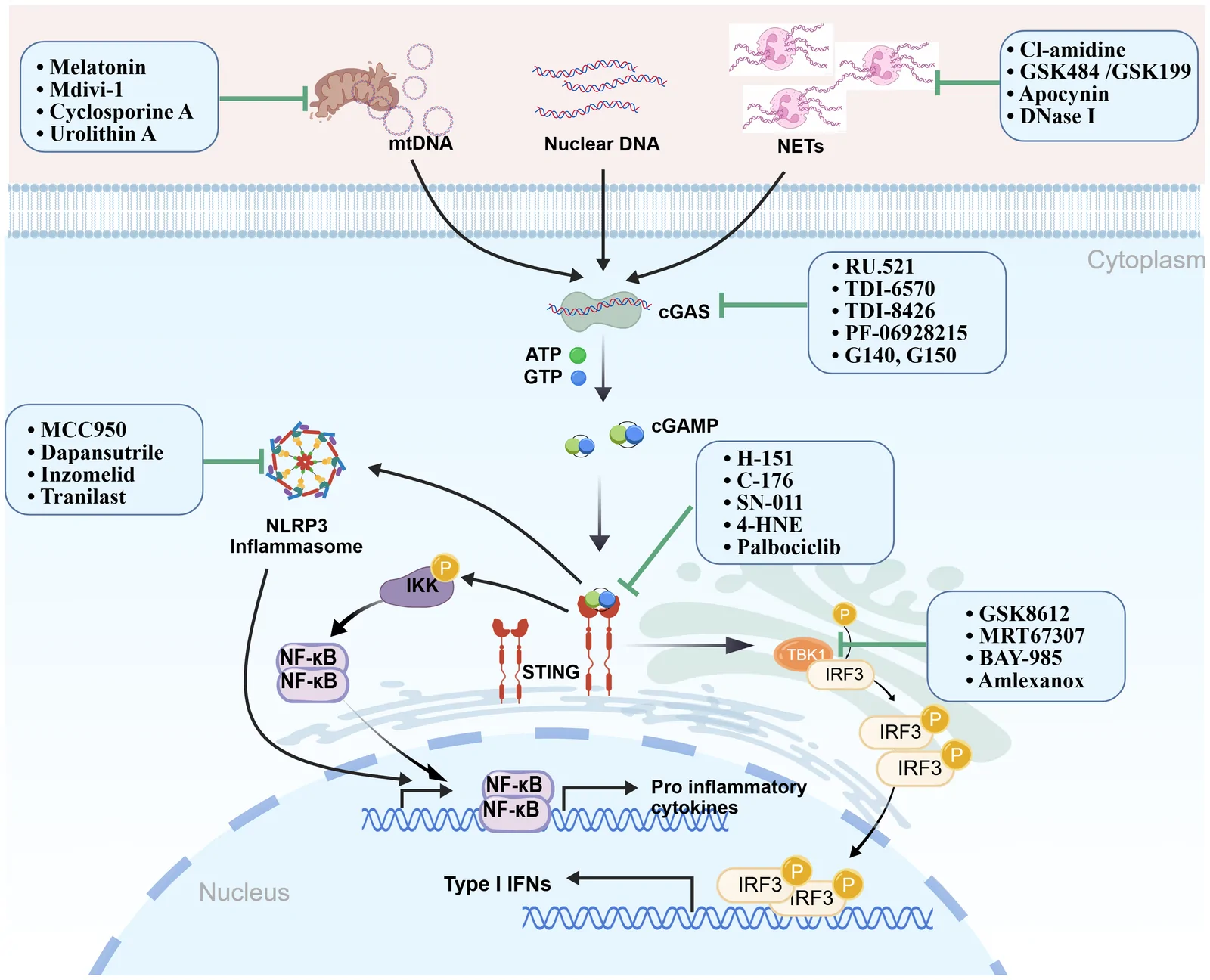
Schematic overview of pharmacological inhibitors targeting multiple nodes of the cGAS–STING signaling cascade. Damaged cells release mitochondrial DNA (mtDNA) and nuclear DNA, while neutrophils produce neutrophil extracellular traps (NETs). These nucleic acids activate the cGAS–STING pathway. A variety of agents can interfere with this inflammatory signaling at distinct steps: compounds including melatonin, Mdivi-1, cyclosporine A and urolithin A suppress mtDNA release; Cl-amidine, GSK484/GSK199, apocynin and DNase I inhibit NET formation or degrade NET-derived DNA; multiple small molecules such as RU.521 and TDI series compounds directly block cGAS activity. STING antagonists (H-151, C-176, SN-011, etc.) restrain STING activation. TBK1 inhibitors including GSK8612, MRT67307 and BAY-985 disrupt downstream IRF3 phosphorylation. In addition, MCC950, dapansutrile and other agents suppress NLRP3 inflammasome activation. Blockade of this signaling axis limits NF-κB-driven pro-inflammatory cytokine production and IRF3-mediated type I IFN transcription.

#### 4.1.3 Human-active STING antagonists

H-151, an indole-based covalent inhibitor optimized from the C-176/C-178 series, retains activity against human STING by alkylating Cys91 and preventing palmitoylation-dependent signaling (59). Notably, H-151 has shown some cytotoxicity at effective concentrations in certain assays, a caveat for chronic CNS dosing (89, 110–112). SN-011 binds the cyclic-dinucleotide (CDN) pocket with higher affinity than endogenous 2’3’-cGAMP and locks the STING dimer in an open, inactive conformation, rescuing Trex1-deficient mice from lethal autoinflammation (89, 112). Because CDN-pocket antagonists act orthosterically rather than covalently, they may offer a cleaner safety profile, but their *in-vivo* CNS pharmacology remains largely uncharacterized (89, 113).

#### 4.1.4 cGAS catalytic inhibitors

RU.521 occupies the cGAS catalytic site with an enzymatic IC_50_ of ~0.11 µM but is substantially more potent against mouse than human cGAS (114). G150 emerged from a human-cGAS-directed screen as a human-selective inhibitor (IC_50_ ~10 nM), with co-crystal structures confirming catalytic-site occupancy (115, 116). Pfizer’s PF-06928215 binds human cGAS with high affinity *in vitro* yet lost activity in cells, most plausibly because millimolar intracellular ATP and GTP outcompete catalytic-site ligands—a general lesson that cellular potency, permeability, and protein binding, rather than biochemical IC_50_, gate CNS utility (114, 115, 117, 118). No cGAS inhibitor has been tested in TBI; the nearest precedent is RU.521 in experimental subarachnoid hemorrhage, where intranasal dosing reduced brain edema and BBB permeability, shifted microglia from an M1- to an M2-like phenotype, and improved neurological outcomes (119, 120).

#### 4.1.5 Indirect pathway modulation

The PERK inhibitor GSK2656157 abrogated ER-stress-driven neuronal STING-IRF3-IFNβ activation after CCI, switching microglia toward an anti-inflammatory phenotype, reducing Th1-cell infiltration, preserving white-matter integrity, and improving affective outcomes (106, 121). Chloroquine, a BBB-penetrant lysosomotropic drug with STING-antagonist properties, reversed cognitive deficits and cortical interferon and inflammatory gene expression when given 1 h after mFPI, phenocopying global STING knockout (30, 107).

### 4.2 Upstream intervention strategies

#### 4.2.1 Mitochondrial protection and blockade of mtDNA release

Because mtDNA is the dominant endogenous cGAS ligand in TBI, preventing its cytosolic release offers an upstream alternative to direct enzymatic inhibition. Pharmacological blockade of MOMP in aged mice reduces sterile inflammation and improves healthspan (77). The hormone FGF21 promoted AMPK-dependent mitophagy, prevented cytosolic mtDNA release, and inhibited cGAS-STING after subarachnoid hemorrhage (99)—a quality-control template directly relevant to TBI.

#### 4.2.2 DNase-mediated clearance of cytosolic DNA

Recombinant DNase I degrades extracellular NET-derived DNA to reduce upstream ligand input for cGAS. In experimental TBI models, DNase I administration mitigates NET-driven neuronal pyroptosis and blood-brain barrier breakdown. Both DNase-I treatment and PAD4 inhibition confer comparable protective phenotypes; critically, such neuroprotection can be abrogated by exogenous 2’3’-cGAMP, a direct STING agonist that bypasses upstream DNA-sensing events (90). However, systemic DNase I has limited CNS penetration, necessitating intrathecal or nanocarrier-delivered formulations.

#### 4.2.3 NET inhibition and PAD4 blockade

The PAD4 inhibitor Cl-amidine dismantled NET formation and attenuated neuronal STING activation and pyroptosis (82). Because NETs accumulate in TBI patient brain and plasma, PAD4 inhibition represents a peripherally actionable strategy that reduces CNS STING ligand load.

### 4.3 Delivery system optimization

Brain exposure is the dominant translational bottleneck. C-176 and H-151 are small lipophilic molecules reported to enter the brain (59, 122); chloroquine is likewise CNS-penetrant. However, most cGAS/STING inhibitors were optimized for peripheral indications, and physicochemical lipophilicity does not guarantee therapeutically relevant unbound brain concentrations (89).

Biological and nanoscale carriers offer more controlled solutions. Angiopep-2-modified extracellular vesicles, which exploit low-density-lipoprotein-receptor-mediated transcytosis, delivered Pgam5 siRNA across the intact BBB and dampened the astrocytic mtDNA-cGAS-STING axis after intracerebral hemorrhage (123). Intranasal, nose-to-brain delivery circumvents the BBB entirely: spherical nucleic acids targeting cGAS-STING signaling reached the CNS after intranasal administration and reshaped the brain immune microenvironment with minimal systemic exposure in a glioblastoma model (124)—a platform readily adaptable to antagonist cargoes.

### 4.4 Repurposed drugs and combination strategies

#### 4.4.1 Repurposed drugs: nucleoside analogs and NAD^+^ precursors

Beyond direct antagonists, several clinically available or development-stage drugs modulate the pathway. Chloroquine is already in clinical use and phenocopied STING knockout in diffuse TBI (30). GSK2656157 is an oncology-derived PERK inhibitor with demonstrated neuroprotective efficacy in TBI (90). FGF21 is in clinical development for metabolic disease and offers mitophagy-enhancing neuroprotection (99).

#### 4.4.2 STING inhibition combined with neuroprotection/BBB repair

Because STING drives NLRP3-dependent pyroptosis after severe TBI, pairing transient STING inhibition with inflammasome or cell-death pathway modulation may achieve additive protection and permit lower doses of each agent. STING inhibition could also be rationally combined with neuroprotective agents or BBB repair strategies to address the multifactorial nature of secondary injury.

## 5 Conclusion

In little more than a decade, the cGAS-STING pathway has progressed from its discovery as an endoplasmic-reticulum adaptor and cytosolic DNA-sensing system (15, 16)to a validated driver of post-traumatic neuroinflammation. Convergent genetic evidence—global and cell-type-specific STING and cGAS knockouts, bone-marrow chimeras, and interferon-pathway mutants—demonstrates that the pathway worsens lesion volume, neuroinflammation, neuronal dysfunction, and cognitive outcome across both focal and diffuse TBI paradigms (32, 34). Pharmacological studies with STING antagonists and agonists establish causality and druggability (46, 118), while human data showing elevated, outcome-correlated cell-free mitochondrial DNA in CSF and serum confirm that the pathway’s ligands are present and measurable in patients. Mechanistically, cGAS-STING sits at a nodal position: it integrates DAMP sensing with microglial activation, peripheral immune recruitment, pyroptotic cell-death programs, and a chronic phase of interferon-rich signaling that drives delayed neurodegeneration—features that collectively explain its outsized influence on both acute outcome and long-term recovery, and that distinguish it from narrower single-effector targets.

Therapeutically, pathway inhibition is rational but must be precision-targeted: delivered in an acute, biomarker-defined window, with human-active compounds, and in a manner that preserves the pathway’s essential antiviral and autophagic functions (38). The immediate priorities are clear—rigorous testing of human-active antagonists across standardized TBI paradigms that include diffuse injury, aging, and both sexes, populations in which pathway activity is amplified and therapeutic windows may differ; development of CSF cGAMP and cf-mtDNA biomarkers for patient stratification and target-engagement readouts; and, ultimately, the first translational and early clinical studies. Meeting these challenges would convert a decade of mechanistic insight into therapeutic reality and position cGAS-STING as one of the most mechanistically grounded and clinically actionable targets in the post-TBI neuroinflammation landscape.

## References

[1] Wenzhe L Boyang X Yuchao G Bimcle R Yue Y . Mitochondrial and ER stress crosstalk in TBI: mechanistic insights and therapeutic opportunities. Front Cell Neurosci. (2025) 19:1697060. doi: 10.3389/fncel.2025.1697060 41480492 PMC12753455

[2] Feng JF Huang XJ Yang XF Gao GY Wang QH He ZH . Traumatic brain injury in China: an update on epidemiology, care pathways, and clinical research. Lancet Neurol. (2026) 25:396–405. doi: 10.1016/s1474-4422(25)00492-2 41864236

[3] Lizon TB Russell R Poovanthodi YA Mangatt S Cabral M Cordero FB . Cell adhesion molecules in traumatic brain injury: orchestrating leukocyte infiltration, BBB disruption, and neuroinflammation. Brain Behav Immun. (2026) 138:106896. doi: 10.1016/j.bbi.2026.106896 42413792 PMC13696158

[4] Toliao JM Lee J Oh H Lee S Conner L Bluml S . Health outcomes following traumatic brain injury: an umbrella review. Neuropsychol Rev. (2026). doi: 10.1007/s11065-026-09704-x 42313356

[5] Menon DK Schwab K Wright DW Maas AI ; Demographics and Clinical Assessment Working Group of the International and Interagency Initiative toward Common Data Elements for Research on Traumatic Brain Injury and Psychological Health. Position statement: definition of traumatic brain injury. Arch Phys Med Rehabil. (2010) 91(11):1637–40. doi: 10.1016/j.apmr.2010.05.017 21044706

[6] Capizzi A Woo J Verduzco-Gutierrez M . Traumatic brain injury: an overview of epidemiology, pathophysiology, and medical management. Med Clin North Am. (2020) 104:213–38. doi: 10.1016/j.mcna.2019.11.001 32035565

[7] Simon DW McGeachy MJ Bayir H Clark RS Loane DJ Kochanek PM . The far-reaching scope of neuroinflammation after traumatic brain injury. Nat Rev Neurol. (2017) 13:171–91. doi: 10.1038/nrneurol.2017.13 28186177 PMC5675525

[8] Corrigan F Mander KA Leonard AV Vink R . Neurogenic inflammation after traumatic brain injury and its potentiation of classical inflammation. J Neuroinflamm. (2016) 13:264. doi: 10.1186/s12974-016-0738-9 27724914 PMC5057243

[9] Chen B Shi QX Nie C Zhao ZP Wang T Zhou Q . Curcumin alleviates oxidative stress, neuroinflammation, and promotes behavioral recovery after traumatic brain injury. Curr Neurovasc Res. (2023) 20:43–53. doi: 10.2174/1567202620666230303144323 36872351

[10] Chen B Shi QX Nie C Zhao ZP Luo L Zhao QJ . Establishment and evaluation of a novel high-efficiency model of graded traumatic brain injury in mice. World Neurosurg. (2021) 154:e7–e18. doi: 10.1016/j.wneu.2021.05.012 33992827

[11] Zhou J Ma S Feng D Tian Y Li L Guo H . C5aR1(+) microglia exacerbate neuroinflammation and cerebral edema in acute brain injury. Neuron. (2026) 114:444–62:e449. doi: 10.1016/j.neuron.2025.10.022 41260217

[12] Marcatti M Labastida JA Tang TZ Ahmad A Payne C Schwartz N . Neuron-derived mitochondrial DNA (mtDNA) activates microglia via the Z-DNA binding protein 1 (ZBP1)-mediated pathway in mild traumatic brain injury. Proc Natl Acad Sci USA. (2026) 123:e2527009123. doi: 10.1073/pnas.2527009123 41926540 PMC13056099

[13] Wang S Shi L Tian Y Wu J Guo F Yu A . Research progress on the relationship between mitochondrial dysfunction and inflammatory response in brain cells following traumatic brain injury: a review. Exp Neurol. (2026) 401:115752. doi: 10.1016/j.expneurol.2026.115752 41933712

[14] Zhang Q Raoof M Chen Y Sumi Y Sursal T Junger W . Circulating mitochondrial DAMPs cause inflammatory responses to injury. Nature. (2010) 464:104–7. doi: 10.1038/nature08780 20203610 PMC2843437

[15] Sun L Wu J Du F Chen X Chen ZJ . Cyclic GMP-AMP synthase is a cytosolic DNA sensor that activates the type I interferon pathway. Science. (2013) 339:786–91. doi: 10.1126/science.1232458 23258413 PMC3863629

[16] Ishikawa H Ma Z Barber GN . STING regulates intracellular DNA-mediated, type I interferon-dependent innate immunity. Nature. (2009) 461:788–92. doi: 10.1038/nature08476 19776740 PMC4664154

[17] Ishikawa H Barber GN . STING is an endoplasmic reticulum adaptor that facilitates innate immune signalling. Nature. (2008) 455:674–8. doi: 10.1038/nature07317 18724357 PMC2804933

[18] Wu J Sun L Chen X Du F Shi H Chen C . Cyclic GMP-AMP is an endogenous second messenger in innate immune signaling by cytosolic DNA. Science. (2013) 339:826–30. doi: 10.1126/science.1229963 23258412 PMC3855410

[19] Ablasser A Goldeck M Cavlar T Deimling T Witte G Rohl I . cGAS produces a 2'-5'-linked cyclic dinucleotide second messenger that activates STING. Nature. (2013) 498:380–4. doi: 10.1038/nature12306 23722158 PMC4143541

[20] White MJ McArthur K Metcalf D Lane RM Cambier JC Herold MJ . Apoptotic caspases suppress mtDNA-induced STING-mediated type I IFN production. Cell. (2014) 159:1549–62. doi: 10.1016/j.cell.2014.11.036 25525874 PMC4520319

[21] Rongvaux A Jackson R Harman CC Li T West AP de Zoete MR . Apoptotic caspases prevent the induction of type I interferons by mitochondrial DNA. Cell. (2014) 159:1563–77. doi: 10.1016/j.cell.2014.11.037 25525875 PMC4272443

[22] Gulen MF Samson N Keller A Schwabenland M Liu C Gluck S . cGAS-STING drives ageing-related inflammation and neurodegeneration. Nature. (2023) 620:374–80. doi: 10.1038/s41586-023-06373-1 37532932 PMC10412454

[23] Sharma V Verma R Sharma P Singh TG . Therapeutic modulation of cGAS-STING pathway in neurodegeneration. Brain Res. (2025) 1864:149784. doi: 10.1016/j.brainres.2025.149784 40517803

[24] Quan S Fu X Cai H Ren Z Xu Y Jia L . The neuroimmune nexus: unraveling the role of the mtDNA-cGAS-STING signal pathway in Alzheimer's disease. Mol Neurodegener. (2025) 20:25. doi: 10.1186/s13024-025-00815-2 40038765 PMC11877805

[25] Liu H Hu C Liu H Gong Z Jiang S Xie J . Mechanistic insights and therapeutic potential of targeting the cGAS-STING pathway in neurodegenerative diseases. J Neuroinflammation. (2026) May 7;23(1):221. doi: 10.1186/s12974-026-03815-1 PMC13317240 42092970

[26] Barrett JP Henry RJ Shirey KA Doran SJ Makarevich OD Ritzel RM . Interferon-beta plays a detrimental role in experimental traumatic brain injury by enhancing neuroinflammation that drives chronic neurodegeneration. J Neurosci. (2020) 40:2357–70. doi: 10.1523/jneurosci.2516-19.2020 32029532 PMC7083281

[27] Abdullah A Zhang M Frugier T Bedoui S Taylor JM Crack PJ . STING-mediated type-I interferons contribute to the neuroinflammatory process and detrimental effects following traumatic brain injury. J Neuroinflamm. (2018) 15:323. doi: 10.1186/s12974-018-1354-7 30463579 PMC6247615

[28] Fritsch LE Ju J Gudenschwager Basso EK Soliman E Paul S Chen J . Type I interferon response is mediated by NLRX1-cGAS-STING signaling in brain injury. Front Mol Neurosci. (2022) 15:852243. doi: 10.3389/fnmol.2022.852243 35283725 PMC8916033

[29] Todd BP Luo Z Larson MJE Ferguson PJ Bassuk AG Newell EA . Type I interferon signaling in microglia drives synaptic engulfment and neuronal loss following traumatic brain injury. Res Sq. (2026). doi: 10.21203/rs.3.rs-9785030/v1 42281997 PMC13252574

[30] Ritzel RM Doran SJ Barrett JP Henry RJ Ma EL Faden AI . Chronic alterations in systemic immune function after traumatic brain injury. J Neurotrauma. (2018) 35:1419–36. doi: 10.1089/neu.2017.5399 29421977 PMC5998829

[31] Lu Z Liu Z Wang C Jiang R Wang Z Liao W . CD300LF(+) microglia impede the neuroinflammation following traumatic brain injury by inhibiting STING pathway. CNS Neurosci Ther. (2024) 30:e14824. doi: 10.1111/cns.14824 38965803 PMC11224125

[32] Henry RJ Ritzel RM Barrett JP Doran SJ Jiao Y Leach JB . Microglial depletion with CSF1R inhibitor during chronic phase of experimental traumatic brain injury reduces neurodegeneration and neurological deficits. J Neurosci. (2020) 40:2960–74. doi: 10.1523/jneurosci.2402-19.2020 32094203 PMC7117897

[33] Fryer AL Abdullah A Mobilio F Jobling A Moore Z de Veer M . Pharmacological inhibition of STING reduces neuroinflammation-mediated damage post-traumatic brain injury. Br J Pharmacol. (2024) 181:3118–35. doi: 10.1111/bph.16347 38710660

[34] Fritsch LE Kelly C Leonard J de Jager C Wei X Brindley S . STING-dependent signaling in microglia or peripheral immune cells orchestrates the early inflammatory response and influences brain injury outcome. J Neurosci. (2024) 44. doi: 10.1523/jneurosci.0191-23.2024 38360749 PMC10957216

[35] Jauhari A Singh T Carlisle DL Friedlander RM . Mitochondrial DNA: a molecular switch driving sterile neuroinflammation. Transl Neurodegener. (2026) 15:5. doi: 10.1186/s40035-026-00540-w 41689141 PMC12903234

[36] Ablasser A Chen ZJ . cGAS in action: expanding roles in immunity and inflammation. Science. (2019) 363. doi: 10.1126/science.aat8657 30846571

[37] Xia L Yan X Zhang H . Mitochondrial DNA-activated cGAS-STING pathway in cancer: mechanisms and therapeutic implications. Biochim Biophys Acta Rev Cancer. (2025) 1880:189249. doi: 10.1016/j.bbcan.2024.189249 39701325

[38] Paul BD Snyder SH Bohr VA . Signaling by cGAS-STING in neurodegeneration, neuroinflammation, and aging. Trends Neurosci. (2021) 44:83–96. doi: 10.1016/j.tins.2020.10.008 33187730 PMC8662531

[39] Li H Cai R Zhou Y Jiang Y Tan S . cGAS-STING signaling in brain aging and neurodegeneration: molecular links and therapeutic perspectives. J Neuroinflamm. (2025) 22:235. doi: 10.1186/s12974-025-03563-8 41094684 PMC12522239

[40] Kranzusch PJ Vance RE . cGAS dimerization entangles DNA recognition. Immunity. (2013) 39:992–4. doi: 10.1016/j.immuni.2013.11.012 24332024

[41] Dvorkin S Cambier S Volkman HE Stetson DB . New frontiers in the cGAS-STING intracellular DNA-sensing pathway. Immunity. (2024) 57:718–30. doi: 10.1016/j.immuni.2024.02.019 38599167 PMC11013568

[42] Chen Q Sun L Chen ZJ . Regulation and function of the cGAS-STING pathway of cytosolic DNA sensing. Nat Immunol. (2016) 17:1142–9. doi: 10.1038/ni.3558 27648547

[43] Cao D Han X Fan X Xu RM Zhang X . Structural basis for nucleosome-mediated inhibition of cGAS activity. Cell Res. (2020) 30:1088–97. doi: 10.1038/s41422-020-00422-4 33051594 PMC7784699

[44] Herzner AM Schlee M Bartok E . The many faces of cGAS: how cGAS activation is controlled in the cytosol, the nucleus, and during mitosis. Signal Transduct Target Ther. (2021) 6:260. doi: 10.1038/s41392-021-00684-3 34244470 PMC8270980

[45] Li T Huang T Du M Chen X Du F Ren J . Phosphorylation and chromatin tethering prevent cGAS activation during mitosis. Science. (2021) 371. doi: 10.1126/science.abc5386 PMC8171060 33542149

[46] Ergun SL Fernandez D Weiss TM Li L . STING polymer structure reveals mechanisms for activation, hyperactivation, and inhibition. Cell. (2019) 178:290–301:e210. doi: 10.1016/j.cell.2019.05.036 31230712

[47] Zhang C Shang G Gui X Zhang X Bai XC Chen ZJ . Structural basis of STING binding with and phosphorylation by TBK1. Nature. (2019) 567:394–8. doi: 10.1038/s41586-019-1000-2 30842653 PMC6862768

[48] Zhao YP Fan G Yin PP Sun S Li N Hong X . Resequencing 545 ginkgo genomes across the world reveals the evolutionary history of the living fossil. Nat Commun. (2019) 10:4201. doi: 10.1038/s41467-019-12133-5 31519986 PMC6744486

[49] Liu S Cai X Wu J Cong Q Chen X Li T . Phosphorylation of innate immune adaptor proteins MAVS, STING, and TRIF induces IRF3 activation. Science. (2015) 347:aaa2630. doi: 10.1126/science.aaa2630 25636800

[50] Wu J Dobbs N Yang K Yan N . Interferon-independent activities of mammalian STING mediate antiviral response and tumor immune evasion. Immunity. (2020) 53:115–26.e115. doi: 10.1016/j.immuni.2020.06.009 32640258 PMC7365768

[51] Yu H Lin L Zhang Z Zhang H Hu H . Targeting NF-kappaB pathway for the therapy of diseases: mechanism and clinical study. Signal Transduct Target Ther. (2020) 5:209. doi: 10.1038/s41392-020-00312-6 32958760 PMC7506548

[52] Balka KR Louis C Saunders TL Smith AM Calleja DJ D'Silva DB . TBK1 and IKKepsilon act redundantly to mediate STING-induced NF-kappaB responses in myeloid cells. Cell Rep. (2020) 31:107492. doi: 10.1016/j.celrep.2020.03.056 32268090

[53] Saitoh T Fujita N Hayashi T Takahara K Satoh T Lee H . Atg9a controls dsDNA-driven dynamic translocation of STING and the innate immune response. Proc Natl Acad Sci USA. (2009) 106:20842–6. doi: 10.1073/pnas.0911267106 19926846 PMC2791563

[54] Dobbs N Burnaevskiy N Chen D Gonugunta VK Alto NM Yan N . STING activation by translocation from the ER is associated with infection and autoinflammatory disease. Cell Host Microbe. (2015) 18:157–68. doi: 10.1016/j.chom.2015.07.001 26235147 PMC4537353

[55] Jeremiah N Neven B Gentili M Callebaut I Maschalidi S Stolzenberg MC . Inherited STING-activating mutation underlies a familial inflammatory syndrome with lupus-like manifestations. J Clin Invest. (2014) 124:5516–20. doi: 10.1172/jci79100 25401470 PMC4348945

[56] Kemmoku H Takahashi K Mukai K Mori T Hirosawa KM Kiku F . Single-molecule localization microscopy reveals STING clustering at the trans-Golgi network through palmitoylation-dependent accumulation of cholesterol. Nat Commun. (2024) 15:220. doi: 10.1038/s41467-023-44317-5 38212328 PMC10784591

[57] Matsumoto K Ni S Arai H Toyama T Saito Y Suzuki T . A non-nucleotide agonist that binds covalently to cysteine residues of STING. Cell Struct Funct. (2023) 48:59–70. doi: 10.1247/csf.22085 36575042 PMC10721953

[58] Mukai K Konno H Akiba T Uemura T Waguri S Kobayashi T . Activation of STING requires palmitoylation at the Golgi. Nat Commun. (2016) 7:11932. doi: 10.1038/ncomms11932 27324217 PMC4919521

[59] Haag SM Gulen MF Reymond L Gibelin A Abrami L Decout A . Targeting STING with covalent small-molecule inhibitors. Nature. (2018) 559:269–73. doi: 10.1038/s41586-018-0287-8 29973723

[60] Shen A Wang X Chen Q Zhang Y Wang F Li Y . Discovery of potent STING inhibitors bearing a difluorobenzodioxol structural motif as potent anti-inflammatory agents. J Med Chem. (2025) 68:8907–32. doi: 10.1021/acs.jmedchem.5c00580 40188441

[61] Konno H Konno K Barber GN . Cyclic dinucleotides trigger ULK1 (ATG1) phosphorylation of STING to prevent sustained innate immune signaling. Cell. (2013) 155:688–98. doi: 10.1016/j.cell.2013.09.049 24119841 PMC3881181

[62] Gui X Yang H Li T Tan X Shi P Li M . Autophagy induction via STING trafficking is a primordial function of the cGAS pathway. Nature. (2019) 567:262–6. doi: 10.1038/s41586-019-1006-9 30842662 PMC9417302

[63] Gulen MF Koch U Haag SM Schuler F Apetoh L Villunger A . Signalling strength determines proapoptotic functions of STING. Nat Commun. (2017) 8:427. doi: 10.1038/s41467-017-00573-w 28874664 PMC5585373

[64] Gaidt MM Ebert TS Chauhan D Ramshorn K Pinci F Zuber S . The DNA inflammasome in human myeloid cells is initiated by a STING-cell death program upstream of NLRP3. Cell. (2017) 171:1110–24.e1118. doi: 10.1016/j.cell.2017.09.039 29033128 PMC5901709

[65] McArthur K Whitehead LW Heddleston JM Li L Padman BS Oorschot V . BAK/BAX macropores facilitate mitochondrial herniation and mtDNA efflux during apoptosis. Science. (2018) 359. doi: 10.1126/science.aao6047 29472455

[66] Riley JS Quarato G Cloix C Lopez J O'Prey J Pearson M . Mitochondrial inner membrane permeabilisation enables mtDNA release during apoptosis. EMBO J. (2018) 37. doi: 10.15252/embj.201899238 30049712 PMC6120664

[67] Jiang J Zuo M Zhao K Ling Z Wu Z Xue D . mtDNA leakage promotes neuron-glia crosstalk to induce epilepsy by cGAS-STING-driven neuroinflammation and serine metabolic reprogramming. Proc Natl Acad Sci USA. (2026) 123:e2522313123. doi: 10.1073/pnas.2522313123 41734071 PMC12956853

[68] Ning X Wang Y Jing M Sha M Lv M Gao P . Apoptotic caspases suppress type I interferon production via the cleavage of cGAS, MAVS, and IRF3. Mol Cell. (2019) 74:19–31.e17. doi: 10.1016/j.molcel.2019.02.013 30878284

[69] Jimenez-Loygorri JI Villarejo-Zori B Viedma-Poyatos A Zapata-Munoz J Benitez-Fernandez R Frutos-Lison MD . Mitophagy curtails cytosolic mtDNA-dependent activation of cGAS/STING inflammation during aging. Nat Commun. (2024) 15:830. doi: 10.1038/s41467-024-45044-1 38280852 PMC10821893

[70] Zhou H Wang X Xu T Gan D Ma Z Zhang H . PINK1-mediated mitophagy attenuates pathological cardiac hypertrophy by suppressing the mtDNA release-activated cGAS-STING pathway. Cardiovasc Res. (2025) 121:128–42. doi: 10.1093/cvr/cvae238 39498806

[71] Simard JM Woo SK Schwartzbauer GT Gerzanich V . Sulfonylurea receptor 1 in central nervous system injury: a focused review. J Cereb Blood Flow Metab. (2012) 32:1699–717. doi: 10.1038/jcbfm.2012.91 22714048 PMC3434627

[72] Walko TD Bola RA Hong JD Au AK Bell MJ Kochanek PM . Cerebrospinal fluid mitochondrial DNA: a novel DAMP in pediatric traumatic brain injury. Shock. (2014) 41:499–503. doi: 10.1097/shk.0000000000000160 24667615 PMC4024373

[73] Kayhanian S Glynos A Mair R Lakatos A Hutchinson PJA Helmy AE . Cell-free mitochondrial DNA in acute brain injury. Neurotrauma Rep. (2022) 3:415–20. doi: 10.1089/neur.2022.0032 36204389 PMC9531878

[74] Mackenzie KJ Carroll P Martin CA Murina O Fluteau A Simpson DJ . cGAS surveillance of micronuclei links genome instability to innate immunity. Nature. (2017) 548:461–5. doi: 10.1038/nature23449 28738408 PMC5870830

[75] Dou Z Ghosh K Vizioli MG Zhu J Sen P Wangensteen KJ . Cytoplasmic chromatin triggers inflammation in senescence and cancer. Nature. (2017) 550:402–6. doi: 10.1038/nature24050 28976970 PMC5850938

[76] Teodoro-Castro B de Faria RC Shashkova EV Malique A Adolph MB Silva LND . A toxic STING-SAMHD1 axis drives replication stress in progeria and cancer cells. Nucleic Acids Res. (2026) 54. doi: 10.1093/nar/gkag614 42306943 PMC13273301

[77] Ichim G Lopez J Ahmed SU Muthalagu N Giampazolias E Delgado ME . Limited mitochondrial permeabilization causes DNA damage and genomic instability in the absence of cell death. Mol Cell. (2015) 57:860–72. doi: 10.1016/j.molcel.2015.05.028 25702873 PMC4352766

[78] Victorelli S Salmonowicz H Chapman J Martini H Vizioli MG Riley JS . Apoptotic stress causes mtDNA release during senescence and drives the SASP. Nature. (2023) 622:627–36. doi: 10.1038/s41586-023-06621-4 37821702 PMC10584674

[79] Harris JL Wang X Christian SK Novikova L Kalani A Hui D . Traumatic brain injury alters the trajectory of age-related mitochondrial change. J Alzheimers Dis. (2024) 97:1793–806. doi: 10.3233/jad-231237 38306050

[80] Lood C Blanco LP Purmalek MM Carmona-Rivera C De Ravin SS Smith CK . Neutrophil extracellular traps enriched in oxidized mitochondrial DNA are interferogenic and contribute to lupus-like disease. Nat Med. (2016) 22:146–53. doi: 10.1038/nm.4027 26779811 PMC4742415

[81] Wang H Wen R Parker E Yang L . A 'Tangled web' in the CNS: unraveling neutrophil extracellular traps in neurological disorders. Mol Neurodegener. (2026). doi: 10.1186/s13024-026-00967-9 42343430 PMC13560142

[82] Wang J Li L Xu J Gheyret D Li K Zhang X . Neutrophil extracellular traps induce endothelial damage and exacerbate vasospasm in traumatic brain injury. Theranostics. (2025) 15:9221–39. doi: 10.7150/thno.115746 40963908 PMC12439479

[83] Zhu Y Xu J Chai Y Li P Liu L Zhang S . Neutrophil extracellular traps aggravate blood-brain barrier disruption via ZBP1/FSP1-mediated ferroptosis after traumatic brain injury. Fluids Barriers CNS. (2025) 23:2. doi: 10.1186/s12987-025-00739-5 41331823 PMC12777371

[84] Wu X Zhou Z Cao Q Chen Y Gong J Zhang Q . Reprogramming of Treg cells in the inflammatory microenvironment during immunotherapy: a literature review. Front Immunol. (2023) 14:1268188. doi: 10.3389/fimmu.2023.1268188 37753092 PMC10518452

[85] Zhang X Xu J Fan Y Shi G Chen B Wang A . Neutrophil extracellular traps aggravate neuronal apoptosis and neuroinflammation via neddylation after traumatic brain injury. Theranostics. (2025) 15:7327–45. doi: 10.7150/thno.111512 40756356 PMC12315821

[86] Packer JM Giammo SG Wangler LM Davis AC Bray CE Godbout JP . Diffuse traumatic brain injury induced stimulator of interferons (STING) signaling in microglia drives cortical neuroinflammation, neuronal dysfunction, and impaired cognition. J Neuroinflamm. (2025) 22:128. doi: 10.1186/s12974-025-03451-1 40307881 PMC12044788

[87] Karve IP Zhang M Habgood M Frugier T Brody KM Sashindranath M . Ablation of Type-1 IFN signaling in hematopoietic cells confers protection following traumatic brain injury. eNeuro. (2016) 3. doi: 10.1523/eneuro.0128-15.2016 27022620 PMC4757777

[88] Chin AC . PERK-STING signaling drives neuroinflammation in traumatic brain injury. J Neurosci. (2020) 40:2384–6. doi: 10.1523/jneurosci.2881-19.2020 32188742 PMC7083527

[89] Decout A Katz JD Venkatraman S Ablasser A . The cGAS-STING pathway as a therapeutic target in inflammatory diseases. Nat Rev Immunol. (2021) 21:548–69. doi: 10.1038/s41577-021-00524-z 33833439 PMC8029610

[90] Barrett JP Henry RJ Vaida M Shirey KA Vogel SN Faden AI . Interferon-beta deficiency selectively modulates chronic microglial pathways after traumatic brain injury. J Neurotrauma. (2026), 8977151261434894. doi: 10.1177/08977151261434894 41913445

[91] Barrett JP Knoblach SM Bhattacharya S Gordish-Dressman H Stoica BA Loane DJ . Traumatic brain injury induces cGAS activation and type I interferon signaling in aged mice. Front Immunol. (2021) 12:710608. doi: 10.3389/fimmu.2021.710608 34504493 PMC8423402

[92] Yang H Wang H Ren J Chen Q Chen ZJ . cGAS is essential for cellular senescence. Proc Natl Acad Sci USA. (2017) 114:E4612–20. doi: 10.1073/pnas.1705499114 28533362 PMC5468617

[93] Gupte R Brooks W Vukas R Pierce J Harris J . Sex differences in traumatic brain injury: what we know and what we should know. J Neurotrauma. (2019) 36:3063–91. doi: 10.1089/neu.2018.6171 30794028 PMC6818488

[94] Yang NS Zhong WJ Sha HX Zhang CY Jin L Duan JX . mtDNA-cGAS-STING axis-dependent NLRP3 inflammasome activation contributes to postoperative cognitive dysfunction induced by sevoflurane in mice. Int J Biol Sci. (2024) 20:1927–46. doi: 10.7150/ijbs.91543 38481801 PMC10929193

[95] Mun Y Kim J Choi YJ Lee BH . cGAS-STING-NF-kappaB axis mediates rotenone-induced NLRP3 inflammasome activation through mitochondrial DNA release. Antioxidants (Basel). (2025) 14. doi: 10.3390/antiox14111276 41300433 PMC12649239

[96] Jindal D Chopra V Garg K Sharma S . Mitochondrial DNA-mediated cGAS-STING activation and its crosstalk with NLRP3 inflammasome in chronic obstructive pulmonary disease. Cytokine Growth Factor Rev. (2026) 90:12–25. doi: 10.1016/j.cytogfr.2026.05.001 42143499

[97] Zhang LM Xin Y Wu ZY Song RX Miao HT Zheng WC . STING mediates neuroinflammatory response by activating NLRP3-related pyroptosis in severe traumatic brain injury. J Neurochem. (2022) 162:444–62. doi: 10.1111/jnc.15678 35892155

[98] Galluzzi L Vitale I Aaronson SA Abrams JM Adam D Agostinis P . Molecular mechanisms of cell death: recommendations of the Nomenclature Committee on Cell Death 2018. Cell Death Differ. (2018) 25:486–541. doi: 10.1038/s41418-017-0012-4 29362479 PMC5864239

[99] Ma Y Liu Z Deng L Du J Fan Z Ma T . FGF21 attenuates neuroinflammation following subarachnoid hemorrhage through promoting mitophagy and inhibiting the cGAS-STING pathway. J Transl Med. (2024) 22:436. doi: 10.1186/s12967-024-05239-y 38720350 PMC11077765

[100] Shaker ME . The contribution of sterile inflammation to the fatty liver disease and the potential therapies. BioMed Pharmacother. (2022) 148:112789. doi: 10.1016/j.biopha.2022.112789 35272137

[101] Kawai C Miyao M Kotani H Minami H Abiru H Tamaki K . Roles of HMGB1 on life-threatening traumatic brain injury and sequential peripheral organ damage. Sci Rep. (2024) 14:21421. doi: 10.1038/s41598-024-72318-x 39271757 PMC11399384

[102] Wang L Zhou Q Chen J Song J Hu W . NSUN6 promotes neuronal axon regeneration after traumatic brain injury by enhancing HMGB1 expression via m5C modification. Brain Res Bull. (2025) 233:111617. doi: 10.1016/j.brainresbull.2025.111617 41197779

[103] Du O Wu YJ Li MY Du JR . The role of HMGB1 in central nervous system (CNS) diseases: mechanisms and therapeutic perspectives. Cytokine. (2026) 198:157099. doi: 10.1016/j.cyto.2025.157099 41455134

[104] Criado-Marrero M Ravi S Barroso D Garza TN Cuestas Torres DM Lessard C . PERK deficiency amplifies molecular, structural, and network vulnerability to repetitive mild traumatic brain injury. Neurobiol Dis. (2026) 228:107526. doi: 10.1016/j.nbd.2026.107526 42435991 PMC13679729

[105] Li L Luo Q Shang B Yang X Zhang Y Pan Q . Selective activation of cannabinoid receptor-2 reduces white matter injury via PERK signaling in a rat model of traumatic brain injury. Exp Neurol. (2022) 347:113899. doi: 10.1016/j.expneurol.2021.113899 34678230

[106] Sen T Saha P Gupta R Foley LM Jiang T Abakumova OS . Aberrant ER stress induced neuronal-IFNbeta elicits white matter injury due to microglial activation and T-cell infiltration after TBI. J Neurosci. (2020) 40:424–46. doi: 10.1523/JNEUROSCI.0718-19.2019 PMC6948950 31694961

[107] Packer JM Bray CE Beckman NB Wangler LM Davis AC Goodman EJ . Impaired cortical neuronal homeostasis and cognition after diffuse traumatic brain injury are dependent on microglia and type I interferon responses. Glia. (2024) 72:300–21. doi: 10.1002/glia.24475 PMC10764078 37937831

[108] Shi G Liu L Cao Y Ma G Zhu Y Xu J . Inhibition of neutrophil extracellular trap formation ameliorates neuroinflammation and neuronal apoptosis via STING-dependent IRE1alpha/ASK1/JNK signaling pathway in mice with traumatic brain injury. J Neuroinflamm. (2023) 20:222. doi: 10.1186/s12974-023-02903-w 37777772 PMC10543875

[109] Li R Xiong Y Ma L Peng C Qi S Gao R . Neutrophil extracellular traps promote macrophage inflammation in psoriasis. Clin Immunol. (2024) 266:110308. doi: 10.1016/j.clim.2024.110308 39002794

[110] Cho Y Oh Y Park J Kwon J Kim TS . STING inhibition by H-151 is associated with amelioration of psoriasis severity via modulation of pathogenic T helper cell subsets. Eur J Pharmacol. (2026) 1015:178564. doi: 10.1016/j.ejphar.2026.178564 41548683

[111] Ozkurt C . Non-covalent engagement of the cGAMP-binding pocket by STING inhibitor H-151: Characterization by molecular docking, Gaussian accelerated molecular dynamics, and MM-GBSA free energy analysis. Comput Biol Chem. (2026) 124:109164. 10.1016/j.compbiolchem.2026.109164 42250479

[112] Hong Z Mei J Li C Bai G Maimaiti M Hu H . STING inhibitors target the cyclic dinucleotide binding pocket. Proc Natl Acad Sci USA. (2021) 118. doi: 10.1073/pnas.2105465118 34099558 PMC8214703

[113] Zhao J Xiao R Zeng R He E Zhang A . Small molecules targeting cGAS-STING pathway for autoimmune disease. Eur J Med Chem. (2022) 238:114480. doi: 10.1016/j.ejmech.2022.114480 35635952

[114] Vincent J Adura C Gao P Luz A Lama L Asano Y . Small molecule inhibition of cGAS reduces interferon expression in primary macrophages from autoimmune mice. Nat Commun. (2017) 8:750. doi: 10.1038/s41467-017-00833-9 28963528 PMC5622107

[115] Hall J Brault A Vincent F Weng S Wang H Dumlao D . Discovery of PF-06928215 as a high affinity inhibitor of cGAS enabled by a novel fluorescence polarization assay. PloS One. (2017) 12:e0184843. doi: 10.1371/journal.pone.0184843 28934246 PMC5608272

[116] Lama L Adura C Xie W Tomita D Kamei T Kuryavyi V . Development of human cGAS-specific small-molecule inhibitors for repression of dsDNA-triggered interferon expression. Nat Commun. (2019) 10:2261. doi: 10.1038/s41467-019-08620-4 31113940 PMC6529454

[117] Shao J Meng Y Yuan K Wu Q Zhu S Li Y . Correction: RU.521 mitigates subarachnoid hemorrhage-induced brain injury via regulating microglial polarization and neuroinflammation mediated by the cGAS/STING/NF-kappaB pathway. Cell Commun Signal. (2024) 22:390. doi: 10.1186/s12964-024-01772-x 39107840 PMC11301847

[118] Wang Y Dou L Gao Y Zhang M Wang Z Wu S . cGAS-STING targeting offers a novel therapeutic paradigm in hemorrhagic stroke. Tissue Cell. (2026) 104:103851. 10.1016/j.tice.2026.103851 42574907

[119] Shao J Meng Y Yuan K Wu Q Zhu S Li Y . RU.521 mitigates subarachnoid hemorrhage-induced brain injury via regulating microglial polarization and neuroinflammation mediated by the cGAS/STING/NF-kappaB pathway. Cell Commun Signal. (2023) 21:264. doi: 10.1186/s12964-023-01274-2 37770901 PMC10537158

[120] Wang Y Yin X Zhang X Zhu X Luo Y Zhao BQ . Inhibition of cGAS reduces brain injury and facilitates neurological recovery via the STING-mediated signaling pathway after germinal matrix hemorrhage in neonatal mice. J Integr Neurosci. (2025) 24:39286. 10.31083/JIN39286 40919625

[121] Sen T Gupta R Kaiser H Sen N . Activation of PERK elicits memory impairment through inactivation of CREB and downregulation of PSD95 after traumatic brain injury. J Neurosci. (2017) 37:5900–11. doi: 10.1523/jneurosci.2343-16.2017 28522733 PMC5473207

[122] Kobritz M Borjas T Patel V Coppa G Aziz M Wang P . H151, a small molecule inhibitor of Sting as a novel therapeutic in intestinal ischemia-reperfusion injury. Shock. (2022) 58:241–50. doi: 10.1097/shk.0000000000001968 35959789 PMC9489661

[123] He J Qin Z Cai Y Liu H Wang T Hu Q . USP11-PGAM5 axis promotes neurotoxic astrocyte reactivity by aggravating the mtDNA-cGAS-STING pathway after intracerebral hemorrhage. Adv Sci (Weinh). (2026) 13:e14283. 10.1002/advs.202514283 PMC12767119 41201111

[124] Mahajan AS Dussold C Kim S Jarvis R Hurley LA Tommasini-Ghelfi S . cGAS-agonistic spherical nucleic acids reprogram the glioblastoma immune microenvironment and promote antitumor immunity. Proc Natl Acad Sci USA. (2025) 122:e2409557122. doi: 10.1073/pnas.2409557122 41183206 PMC12626011

